# Medicinal waterbirds in the traditional healthcare system: an assessment of biodiversity–cultural linkages in Eastern Khyber Pakhtunkhwa, Pakistan

**DOI:** 10.1186/s13002-022-00554-4

**Published:** 2022-08-27

**Authors:** Qaisar Rahman, Muhammad Sajid Nadeem, Muhammad Umair, Muhammad Altaf, Jian Ni, Arshad Mahmood Abbasi, Muhammad Azhar Jameel, Andrea Pieroni, Muhammad Haroon Hamed, Sana Ashraf, Tasnim Sadaf

**Affiliations:** 1 Department of Zoology, Wildlife and Fisheries, PMAS-Arid Agriculture University, Rawalpindi, 46300 Pakistan; 2 College of Chemistry and Life Sciences, Zhejiang Normal University, Jinhua, 321004 China; 3 Department of Forestry, Range and Wildlife Management, The Islamia University of Bahawalpur, 63100 Bahawalpur, Pakistan; 4 Department of Environment Sciences, COMSATS University Islamabad, Abbottabad Campus, 22060 Abbottabad, Pakistan; 5 University of Gastronomic Sciences, Piazza Vittorio Emanuele II 9, 12042 Pollenzo, Italy; 6 Department of Medical Analysis, Tishk International University, 4401 Erbil, Iraq; 7 Department of Zoology, Wildlife and Fisheries, University of Agriculture, Faisalabad, 38000 Pakistan; 8 Department of Zoology, University of Lahore, Sargodha, 40100 Pakistan; 9 Department of Wildlife and Ecology, University of Veterinary and Animal Sciences, 54000 Lahore, Pakistan

**Keywords:** Ethno-ornithology, Waterbirds, Pakistan, Principal component analysis, Chord diagram

## Abstract

**Background:**

Eastern Khyber Pakhtunkhwa is home to a vast range of medicinal and edible waterbird species due to its diverse geographical environment. Waterbird species have been used for various ailments and cultural practices since ancient times, while ethno-pharmacological applications and cultural uses of waterbird species in this area have seldom been documented. This study is the first ethnomedicinal and cultural assessment of waterbird species, and the first compilation and listing of all known data on these species in Eastern Khyber Pakhtunkhwa, Pakistan.

**Methods:**

Interviews and questionnaires were used to collect data from native respondents (*N* = 100). To analyze the data, principal component analysis (PCA), relative frequency of citation (RFC), fidelity level (FL%), relative popularity level (RPL), rank order priority, and similarity index were used.

**Results:**

In total, 64 waterbird species were utilized in cultural practices, of which 40 species are used to cure different infectious and chronic diseases such as cold, cough, flu, fever, respiratory disorders, asthma, TB, gastric ulcers, kidney stones, male impotency, obesity, paralysis, piles, cancer, arthritis, body pain, and weakness. PCA showed significant differences in the use of waterbird species among the local inhabitants of the study area, separated along the axis-2 (*p* < 0.05). The FL% of waterbird species varied from 12 to 100%. 100% FL was analyzed for four waterbird species, i.e., *Charadrius mongolus* (cold), *Gallicrex cinerea* (asthma), *Anas platyrhynchos* (cancer), and *Esacus recurvirostris* (body weakness). In this study, Mallard (*Anas platyrhynchos*) was the most popular species used in the healthcare system of Eastern Khyber Pakhtunkhwa, with high RFC (4.06), FL% (100), and RPL (1.0) values.

**Conclusion:**

We concluded that waterbird species are more used for medicine and food purposes in the study area. However, in vitro/in vivo assessment of biochemical activities of waterbird species with a maximum FL% might be significant to produce novel drugs. Recent research shows important ethno-ornithological information about native people and their links with waterbird species, which might be helpful for the sustainable use of waterbird diversity in the research area.

## Introduction

Ethno-ornithology is a natural scientific approach that explains the relationship between people’s knowledge and the use of birds in their culture [1–5]. It is essential in ethno-ornithological research that a bird's presence, movements, habits, and associated local knowledge be recorded correctly and in a way that all people can access the information [6]. In several human ethnic communities, bird species constitute the major source of protein [7–9] and fats [10]; they are used in medicine, commercial as well as in folklore [11, 12]. In Pakistan, herbivores, granivores, frugivores, and omnivore species (which do not eat dead animals) were edible and used as food [11]. Birds and parts of birds are also known for their healing properties around the world [13]. Bird’s highest percentages of recipes are used to treat respiratory disorders, body weakness, gastrointestinal problems, and skin infections [11]. For example, the insides of a Neotropic Cormorant, *Phalacrocorax brasilianus*, spread on the chest, were an antidote for a person suffering from asthma [14].

Pakistan currently has a diverse and dense bird population [1, 15–23] with almost 668 known species [24, 25], and a number of waterbird species are utilized by societies [2, 11, 12]. Very old associations have been developed between waterbirds and human societies, and these waterbird species are documented in the thoughts of human societies in various ways [26]. Waterbird species are generally documented in terms of their roles as entertainment, commercial, pets, magic, medicine [1, 2, 5, 8], or sources of food [3, 11], though these birds have other significant symbolic and medicinal relationships with humans [27]. Birds are among the fauna commonly utilized in ethnomedicine in Pakistan [1, 3–5, 11, 19, 28] and other countries on this planet [29, 30]. Anatidae and other waterbirds have cultural significance in many parts of the world [31]. Cultural uses of waterbird species (i.e., food, hunting, medicine, entertainment, religious practice, and trade) may promote beliefs and behaviors that help to conserve these species [32, 33]. However, if they are practiced unsustainably, or affected by commercialization or other political and economic factors, they may negatively affect or even endanger these species [33]. The use of waterbird species in traditional medicine and for cultural purposes by local communities must also be considered in relation to other factors, such as changes in climate and habitat [26, 34]. Because of anthropogenic influences, wetlands are rapidly diminishing biodiversity and risking freshwater supplies for waterbird habitats [35]. Waterbirds have been identified as being extremely sensitive to climate change [36] and even more vulnerable to changes in land cover or human-engineered land use [37]. Studies have also demonstrated that agricultural runoff into wetlands can reduce waterbird populations by contaminating the water with pesticides [38–40].

Waterbirds are major players in the aquatic ecosystem theater, providing a variety of important ecosystem services [33, 41]. Waterbirds can help to preserve the diversity of other species by controlling pests, acting as effective bio-indicators of environmental conditions, and responding as indicator species for potential disease outbreaks [33, 42]. They also provide essential provisioning (eggs, meat, feathers, etc.) and cultural services to both modern and indigenous societies [43, 44]. Various waterbird species are currently used for ethnomedicine, folklore drugs, and nanomedicine. However, there is an urgent need to investigate the ecological, cultural, social, and ethnomedicine aspects of waterbird species’ use for sustainable management and conservation of bio-resources. The main purpose of this study was to (i) document the cultural uses of waterbirds as well as traditional knowledge about the medicinal uses of waterbird species, and (ii) collect data on traditional therapies for a variety of diseases, including parts used, preparation methods, and applications, to preserve the traditional knowledge of medicinal uses against various human ailments.

## Materials and methods

### Description of the study area

**Fig. 1:**
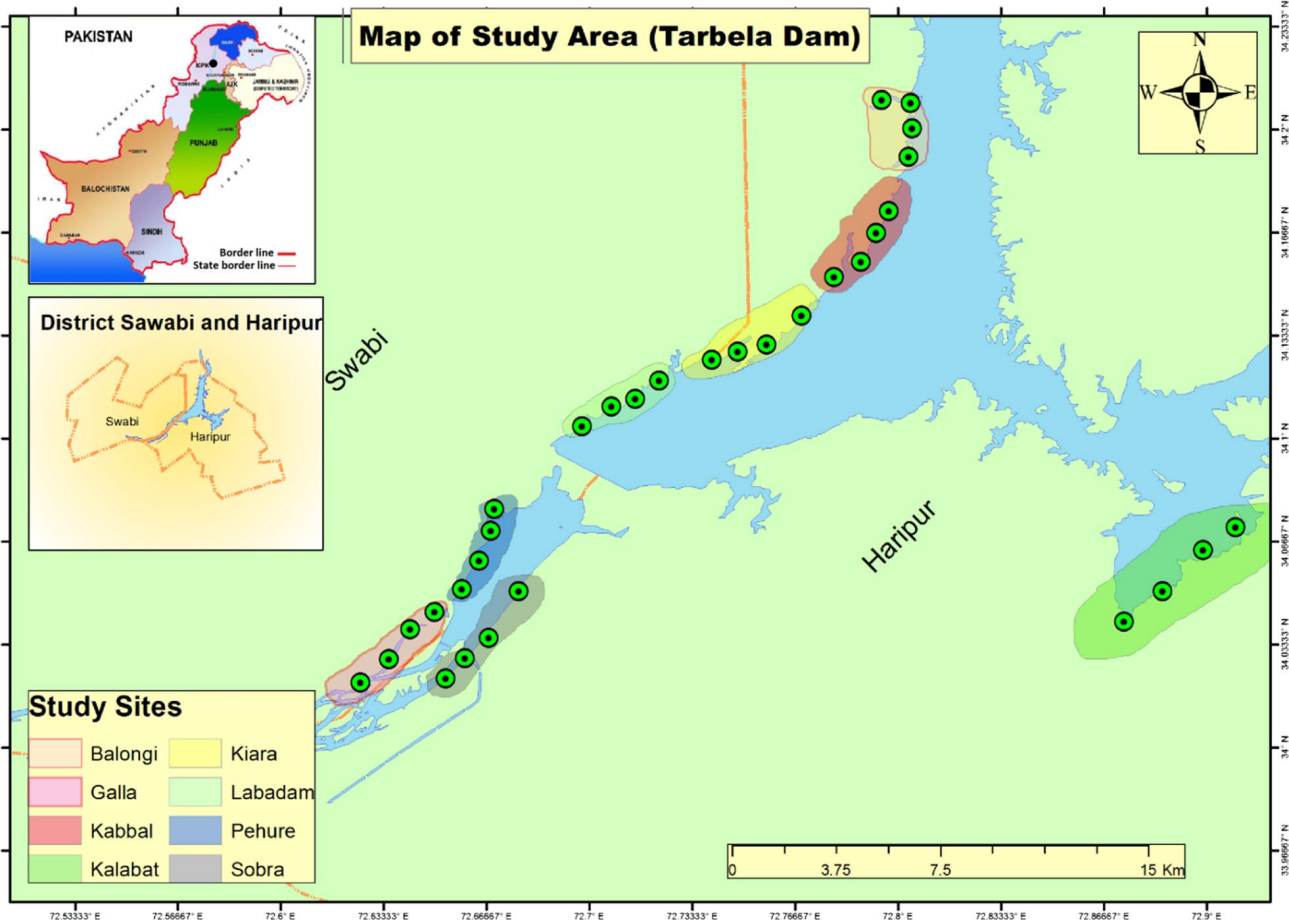
Map of the study area showing the sampling sites in Eastern KPK, Pakistan

Swabi and Haripur, the most populous districts, are situated between the Indus River and Kabul River in Eastern KPK (Fig. 1). The Tarbela Dam is present on the Indus River (34° 7′ 35″ North, 72° 48′ 37″ East) in Haripur District, KPK, about 50 km northwest of Islamabad. The Indus is the largest flow through the Karakorum and Himalayan Mountains and passes from Tarbela Dam. The flow of water is higher in the period of monsoon compared with other seasons. Most of the area (78.0%) of the district is mountainous, and the rest (21.0%) is plain dry land. The climate of the study area is hot in summer (April to September) with maximum temperatures between 38 and 46 °C. June is the hottest month, and winters are relatively cold with minimum temperatures between 3 and 14 °C. The average rainfall recorded was 1026 mm/annum. The humidity is relatively high throughout the year [45]. Yousafzai are in majority and other tribes are Razar, Utman kheil, Jadoon, Khattak, and Hindkyan (Hindko speakers). Most of the people are directly or indirectly involved in agriculture [46]. According to a literature review, 60 percent of the population in this area derives their income from the forests [47]. A total of 29 mammalian species, 9 species of amphibians, 26 species of reptiles, and 89 species of waterbird including 68 migratory waterbird species have been documented from Tarbela Dam in Eastern Khyber Pakhtunkhwa, Pakistan [45, 48–52]. The unique characteristics of this region, such as significant temperature, altitude, geography, soil type, and moisture variation, make it extremely valuable from a medicinal point of view.

### Ethno-ornithology documentation and identification

During the field survey, the main focus was on quantifying, exploring, and comparing ethno-ornithological knowledge among different rural communities in the study area. The data were collected from the selected sub-areas such as Kalabat town, Kiara, Labadam, Pehur, Sobera, Balongi, Kabbal, and Gala from March 2019 to February 2020 (Fig. 1). Data on ethnomedicinal applications of waterbird species were obtained through semi-structured interviews and discussions using the methods previously described [53, 54]. The study's principal author is a local resident who visited the various places (high and low altitude) in the region with a photographer. Prior to collecting data, verbal consent was obtained from all local respondents after briefing the research objectives. Ethical guidelines of the International Society of Ethno-biology (http://www.ethnobiology.net/) were strictly followed. Questionnaires and semi-structured interviews were conducted with 100 informants, i.e., farmers, teachers, herdsmen, hunters, and traditional health practitioners (THPs). Informants were selected based on their traditional knowledge on the medicinal and cultural importance of bird species. Personal information of informants, local names of waterbird species, cultural importance of waterbird species, and ethno-pharmacological uses of waterbirds were all included in the questionnaires. Books of “Birds of Pakistan” were noted for correct identification of waterbird species of the region [50, 51]. The diversity of waterbirds in the study area was estimated through the linear count survey method, and the direct (i.e., physical count mean direct observation with camera and naked eye and voices) and indirect (i.e., nests and group questionnaire surveys or meetings) methods were utilized [55]. Moreover, the species’ scientific names were checked and corrected by using the Global Biodiversity Information Facility (https://www.gbif.org) and Catalogue of Life (https://www.catalogueoflife.org).

### Ethical approval

The proposed research on birds, especially waterbird species, was duly approved by the Institutional Ethical Committee (IEC), PMAS-Arid Agriculture University Rawalpindi, Pakistan (Ref No. PMAS-AAUR/IEC/15), focusing on the ethnomedicinal research and intellectual property rights of informants before the field survey. In addition, the ethical guidelines and rules of the International Society of Ethno-biology (ISE) (http://www.ethnobiology.net/) were strictly followed.

### Quantitative analysis

The waterbird data were observed using six various indices: “Principal Component Analysis,” “Relative Frequency of Citation,” “Fidelity Level,” “Relative Popularity Level,” “Rank Order Priority,” and “Similarity Index.”

### Frequency of citation (FC)

“FC” presents the number of local participants who cited the ethnomedicinal uses of each waterbird species [11, 56].

### Relative frequency of citation (RFC)

“RFC” presents the significance of all species from the region [57, 58]. RFC is calculated by dividing the number of informers citations of a particular waterbird species (FC) by the whole number of respondents in the study area (*N*) [53, 59]. “RFC” was analyzed by Eq. (1) as follows:

1 $${\text{RFC}} = \frac{{{\text{FC}}}}{N}\;(0 < {\text{RFC}} < 1).$$

### Fidelity level (FL%)

FL is the percentage of informers declaring the uses of an exacting kind of particular number of ethnomedicinal uses of diversity of birds through informer in the region. The FL index was analyzed utilizing Eq. (2) as follows [56, 60]:

2 $${\text{FL}}\% = \frac{{N_{p} }}{N} \times 100$$

where “*N*_*p*_^**”**^ is the informers’ number for exacting types of ethnomedicinal uses of fauna and “*N*” is the total informers that noted the fauna for uses. A high “FL” index documented the significance and a high number of uses of fauna for ethno-cultural use by the informers of the region.

### Relative popularity level (RPL)

The “RPL” is the proportion of the ethno-cultural use number by notifying fauna and the sum number of informers for sickness. Though waterbird species with similar “FL” values, however, were recognized by various informers, that may be different in their curing capability. A scale was as a result created as follows: All the waterbirds documented were separated into “popular” as well as “unpopular” factions. The “RPL” presumes a value “zero” and “one” with 1.0 being the total popularity of a particular waterbird species for major sickness and “zero” for no sickness cured by a particular species. While all livings are uniformly important for major sicknesses, a “popularity index” would be at a maximum of “one” and reduce toward “zero” as the relative popularity of the waterbird species deviates away from the popular part. For popular waterbirds, the “RPL” value is logically preferred to equivalent “1.0.” For waterbirds within “unpopular group,” the “RPL” value is lower than “one.” The “RPL” value may be resolute for each particular species in accordance with its accurate place on the grid [56, 61].

### Rank order priority (ROP)

The “ROP” of the waterbirds is utilized to suitably rank the waterbirds with “FL” values and “RPL” values utilized as correction feature. The “ROP” is derived from “FL” multiplying with “RPL.” The “ROP” value was analyzed by Eq. (3) [56, 61].

3 $${\text{ROP}} = {\text{RPL}} \times {\text{FL}}$$

### Similarity index (SI)

SI is collected by the following formula (4):

4 $${\text{SI}} = \frac{{S_{{\text{a}}} }}{{T_{{\text{a}}} }}\;(0 < {\text{SI}} < 1)$$

Note that *S*_a_ = similar documented ailment in the previous and present study; *T*_a_ = total documented ailment in the present study.

### Statistical analysis

We used multivariate ordination principal component analysis (PCA) to discover patterns of the ethno-cultural and ethnomedicinal uses of waterbird species by using the ethno-data variables. A one-way ANOVA was performed to check the significance of PCA scores. The contribution of different part use and mode of application were displayed in chord diagrams using “circlize package (24)” in R software 3.6.1 [62]. All graphical data analyses were performed using Microsoft Excel 2010 (Microsoft, Redmond, WA, USA), R software 3.6.3, and PAST 3.20 [63].

## Results

### Demographic features of respondents

**Fig. 2:**
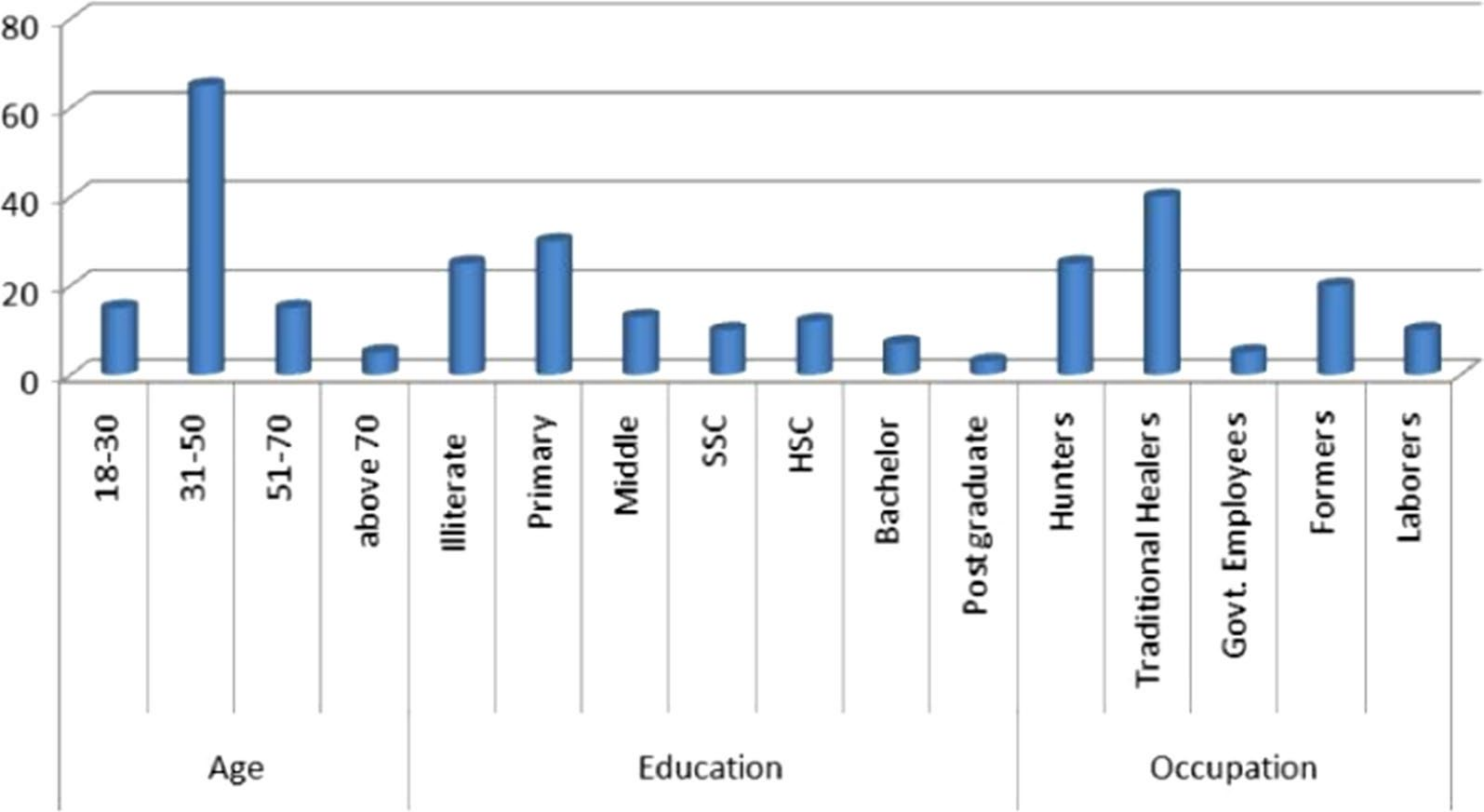
Number of study participants in Eastern KPK, Pakistan. Respondents of different age, occupation, and education were interviewed

In total, 100 informers were selected from 18 to 75 years of age (Fig. 2). However, the maximum number of respondents (*n* = 64) was between the ages of 31 and 50 years. Approximately 75 respondents were literate, viz. primary (*n* = 30), middle (*n* = 13), secondary school certificate (SSC) (*n* = 10), higher school certificate (HSC) (*n* = 35), bachelor (*n* = 7), and post-graduate (*n* = 3). 79% of the respondents were from rural areas. The older informers have important traditional ecological knowledge as compared to the younger ones. Selected informants belong to different occupations such as hunters, traditional health practitioners (THPs), government employees, formers, and laborers (Fig. 2).

### Taxonomic classification

**Fig. 3:**
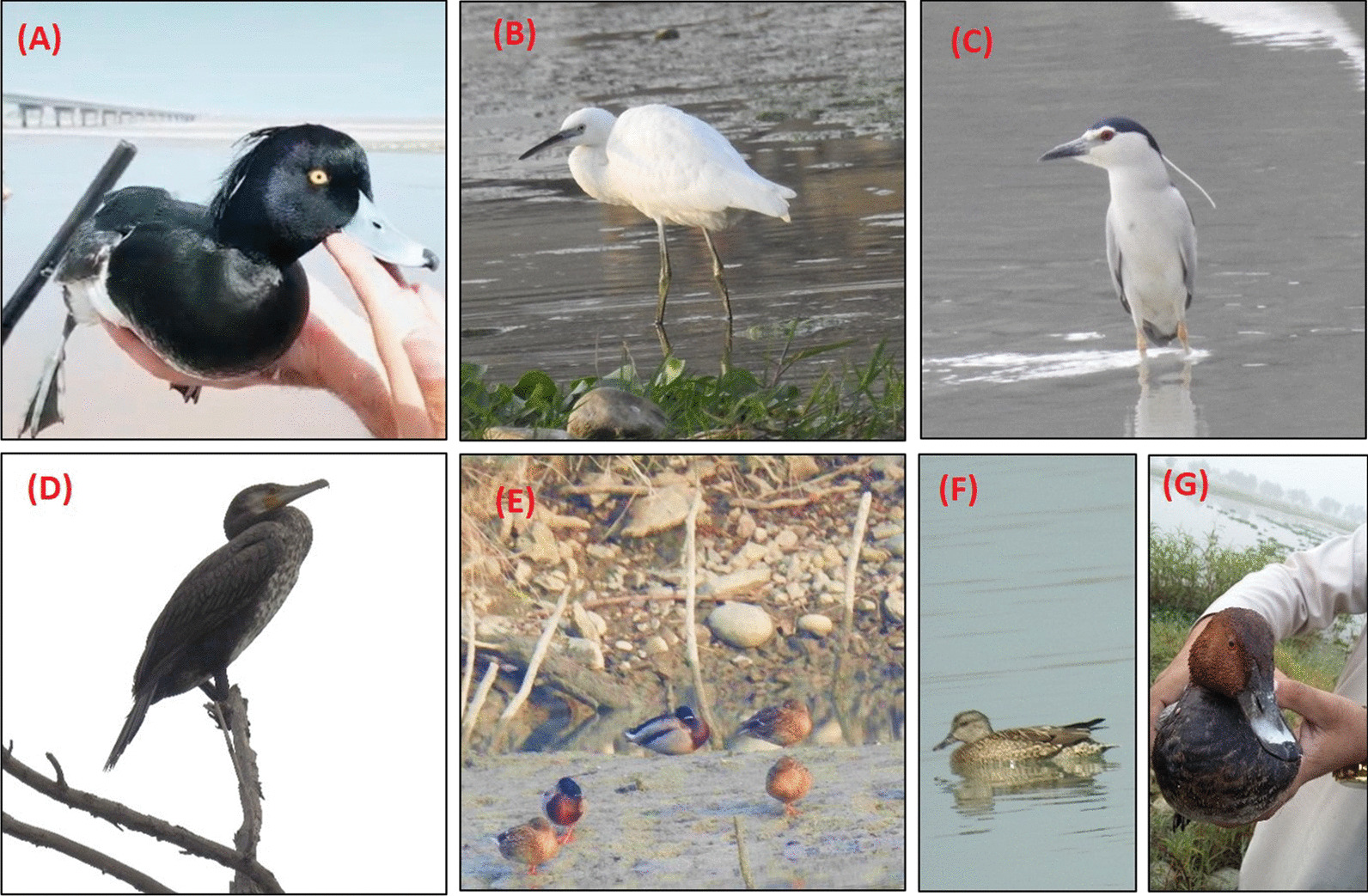
Some important waterbirds of the study area. **A** Tufted duck, **B** little white egret, **C** black-crowned night heron, **D** great cormorant, **E** mallard, **F** gadwall and **G** common pochard

In total, 64 waterbird species from 9 orders and 17 families were reported (Table 1 and Fig. 3). Anseriformes was the most dominant order with 18 species, followed by Charadriiformes (16 species), Pelecaniformes (8 species), Passeriformes (7 species), Gruiformes (5 species), Coraciiformes (4 species), Laridae, Podicipediformes, and Suliformes (2 species each) (Table 1).

**Table 1:** Ethno-ornithological applications among the local people of study area

Sr. No.	Scientific name Common name (local name)	Family (order)	FC	MD	FD	SPS	HN	EX	OR
1	*Ceryle rudis* (Linnaeus, 1758) Pied Kingfisher (Mahe Khawarak)	Alcedinidae (Coraciiformes)	16	0	0	2	0	0	15
2	*Alcedo atthis* (Linnaeus, 1758) Common Kingfisher (Shentagh)	Alcedinidae (Coraciiformes)	15	0	0	2	0	0	15
3	*Anas strepera* Linnaeus, 1758 Gadwall (Khar sari batha/gadwall)	Anatidae (Anseriformes)	81	39	55	11	79	6	26
4	*Anas crecca* Linnaeus, 1758 Green-winged Teal (Warri choraki)	Anatidae (Anseriformes)	79	42	60	11	79	10	26
5	*Anas platyrhynchos* Linnaeus, 1758 Mallard (Sheen sari Batha)	Anatidae (Anseriformes)	85	47	78	26	84	17	30
6	*Anas acuta* Linnaeus, 1758 Northern Pintail (Laki mar Batha)	Anatidae (Anseriformes)	78	39	65	11	78	3	23
7	*Anas clypeata* Linnaeus, 1758 Northern Shoveler (Shabli)	Anatidae (Anseriformes)	70	36	47	11	68	13	20
8	*Netta rufina* (Pallas, 1773) Red-crested Pochard (Shabar)	Anatidae (Anseriformes)	74	14	32	11	69	0	13
9	*Aythya ferina* (Linnaeus, 1758) Common Pochard (Sor-sari Batha)	Anatidae (Anseriformes)	80	27	72	17	76	13	15
10	*Aythya fuligula* (Linnaeus, 1758) Tufted Duck (Ziar Stargi Batha)	Anatidae (Anseriformes)	42	9	39	3	25	0	17
11	*Anas querquedula* Linnaeus, 1758 Garganey (Kar kari/Gargany)	Anatidae (Anseriformes)	67	45	63	0	45	7	12
12	*Anas Penelope* Linnaeus, 1758 Eurasian Wigeon (Seti mar)	Anatidae (Anseriformes)	70	48	60	5	68	5	11
13	*Bucephala clangula* (Linnaeus, 1758) Common Goldeneye (Ziar Stargi Batha/ Zangli Charga)	Anatidae (Anseriformes)	55	17	51	0	8	0	0
14	*Tadorna tadorna* (Linnaeus, 1758) Common shelduck (Spena Batha)	Anatidae (Anseriformes)	64	39	64	3	43	0	15
15	*Tadorna ferruginea* (Pallas, 1764) Ruddy Shelduck (Sorhab)	Anatidae (Anseriformes)	67	36	67	5	43	3	15
16	*Aythya nyroca* (Güldenstädt, 1770) Ferruginous Duck (Seti mar wari Batha)	Anatidae (Anseriformes)	38	32	37	3	25	2	12
17	*Mergellus albellus* (Linnaeus, 1758) Smew (Spena Batha)	Anatidae (Anseriformes)	36	26	36	3	15	0	7
18	*Anser anser* (Linnaeus, 1758) Greylag Goose (Warri margabi)	Anatidae (Anseriformes)	73	9	45	7	73	17	36
19	*Anser albifrons* (Scopoli, 1769) Great White-fronted Goose (Ghatti margabi)	Anatidae (Anseriformes)	73	9	45	7	73	17	36
20	*Mergus merganser* Linnaeus, 1758 Common Merganser (Torsari Bathe)	Anatidae (Anseriformes)	48	27	47	0	32	3	15
21	*Mesophoyx intermedia* (Wagler, 1829) Intermediate Egret (Dermeiani Bagle)	Ardeidae (Pelecaniformes)	18	0	0	7	0	0	6
22	*Egretta garzetta* (Linnaeus, 1766) Little Egret (Warri-spene Bagle)	Ardeidae (Pelecaniformes)	8	0	0	7	0	0	5
23	*Ardea cinerea* Linnaeus, 1758 Grey Heron (Khari Bagle)	Ardeidae (Pelecaniformes)	18	0	0	9	0	0	16
24	*Nycticorax nycticorax* (Linnaeus, 1758) Black-crowned Night Heron (Taj-wala Bagle)	Ardeidae (Pelecaniformes)	17	0	0	9	0	0	16
25	*Botaurus stellaris* (Linnaeus, 1758) Eurasian Bittern (Eurasian Bagla)	Ardeidae (Pelecaniformes)	10	0	0	3	0	0	9
26	*Ardeola grayii* (Sykes, 1832) Indian Pond Heron (Mashriqi Bagla)	Ardeidae (Pelecaniformes)	13	0	0	3	0	0	13
27	*Ardea alba* Linnaeus, 1758 Great Egret (Gaati Bagla)	Ardeidae (Pelecaniformes)	7	0	0	7	0	0	1
28	*Bubulcus ibis* (Linnaeus, 1758) Cattle Egret (Zenawaro Bagla)	Ardeidae (Pelecaniformes)	10	0	0	5	0	0	9
29	*Esacus recurvirostris* (Cuvier, 1829) Great Stone-curlew (Ghatee Kharari)	Burhinidae (Charadriiformes)	4	5	0	0	2	0	4
30	*Pluvialis squatarola* (Linnaeus, 1758) Grey Plover (Kharari)	Charadriidae (Charadriiformes)	14	5	12	0	1	0	4
31	*Charadrius alexandrinus* Linnaeus, 1758 Kentish or Snowy Plover (Speni Kharari)	Charadriidae (Charadriiformes)	10	5	5	0	2	0	3
32	*Vanellus indicus indicus* (Boddaert, 1783) Red-wattled Lapwing (Sor titara)	Charadriidae (Charadriiformes)	16	0	0	9	0	0	16
33	*Vanellus malabaricus* (Boddaert, 1783) Yellow-wattled Lapwing (Zair titara)	Charadriidae (Charadriiformes)	18	0	0	9	0	0	16
34	*Charadrius mongolus* Pallas, 1776 Lesser Sand Plover (Warri Kharari)	Charadriidae (Charadriiformes)	5	5	0	0	1	0	1
35	*Ciconia nigra* (Linnaeus, 1758) Black stork (Tor Zanari)	Ciconiidae (Ciconiiformes)	23	9	23	5	13	0	13
36	*Ciconia ciconia* Linnaeus, 1758 White stork (Spen Zanari)	Ciconiidae (Ciconiiformes)	28	7	28	5	13	0	13
37	*Grus grus* (Linnaeus, 1758) Common Crane (Zanrai)	Gruidae (Gruiformes)	38	0	23	0	37	11	26
38	*Haematopus ostralegus* Linnaeus, 1758 Eurasian Oystercatcher (Mahe Khawarak)	Haematopodidae (Charadriiformes)	15	0	0	0	0	0	13
39	*Riparia riparia* (Linnaeus, 1758) Sand Martin (Khar Totakarki)	Hirundinidae (Passeriformes)	21	0	0	21	0	0	3
40	*Cecropis daurica* (Laxmann, 1769) Red-rumped Swallow (Sor Totakarki)	Hirundinidae (Passeriformes)	5	0	0	5	0	0	2
41	*Ptyonoprogne obsoleta* (Cabanis, 1850) Pale Crag Martin (Beranga Totakarki)	Hirundinidae (Passeriformes)	22	0	0	21	0	0	2
42	*Larus ridibundus* (Linnaeus, 1766) Common Black-headed Gull (Ghatti Torsari Bagle)	Laridae (Charadriiformes)	5	0	0	3	0	0	2
43	*Larus marinus* Linnaeus, 1758 Great Black-backed Gull (Ghatti obo Bagle)	Laridae (Charadriiformes)	5	0	0	3	0	0	3
44	*Sterna acuticauda* (Gray, 1832) Black-bellied tern (Totakarki)	Laridae (Charadriiformes)	26	0	0	21	0	0	26
45	*Larus cachinnans* Pallas, 1811 Caspian Gull (Obo Bagla)	Laridae (Charadriiformes)	8	0	0	2	0	0	7
46	*Larus fuscus* Linnaeus, 1758 Lesser Black-backed Gull (Warri-torsari Bagle)	Laridae (Charadriiformes)	14	0	0	3	0	0	14
47	*Sterna hirundo* Linnaeus, 1758 Common Tern (Kaaz/Babozi)	Laridae (Charadriiformes)	13	1	0	13	7	0	8
48	*Motacilla alba* Linnaeus, 1758 White Wagtail (Spina Chinchi lakai)	Motacillidae (Passeriformes)	10	0	0	1	0	0	6
49	*Motacilla cinerea* Tunstall, 1771 Grey Wagtail (Chinchi Lakai/Tan Tanai)	Motacillidae (Passeriformes)	3	0	0	1	0	0	3
50	*Pelecanus onocrotalus* Linnaeus, 1758 Great White Pelican (Kotanara)	Pelecanidae (Pelecaniformes)	80	79	0	0	13	17	21
51	*Microcarbo niger* (Vieillot, 1817) Little Cormorant (Warri-tore elli)	Phalacrocoracidae (Suliformes)	55	36	54	0	46	0	15
52	*Phalacrocorax carbo* (Linnaeus, 1758) Great Cormorant (Ghati-tore elli)	Phalacrocoracidae (Suliformes)	54	36	54	0	46	0	15
53	Podiceps cristatus (Linnaeus, 1758) Great Crested Grebe (Ghati grab)	Podicipedidae (Podicipediformes)	57	21	57	11	35	3	13
54	*Tachybaptus ruficollis* (Pallas, 1764) Little Grebe (Warri greb)	Podicipedidae (Podicipediformes)	57	21	57	11	35	3	13
55	*Porphyrio porphyrio* (Linnaeus, 1758) Purple swamphen (Jamani Charga)	Rallidae (Gruiformes)	15	5	0	0	0	0	0
56	*Rallus aquaticus* Linnaeus, 1758 Water Rail (Khawar chargai)	Rallidae (Gruiformes)	10	3	0	0	0	0	8
57	*Fulica atra* Linnaeus, 1758 Eurasian Coot (Jal Kokar)	Rallidae (Gruiformes)	76	54	76	4	72	3	14
58	*Gallinula chloropus* (Linnaeus, 1758) Common Moorhen (Obo Charga)	Rallidae (Gruiformes)	8	2	0	0	0	0	7
59	*Gallicrex cinerea* (Gmelin, 1789) Watercock (Zanglai Charga)	Rallidae (Gruiformes)	7	7	0	0	0	0	6
60	*Himantopus himantopus* (Linnaeus, 1758) Black-winged stilt (Tor tetari)	Recurvirostridae (Charadriiformes)	8	3	0	0	0	0	5
61	*Recurvirostra avosetta* Linnaeus, 1758 Pied avocet (Loi mahoki tetara)	Recurvirostridae (Charadriiformes)	20	2	11	0	9	0	17
62	*Calidris temminckii* (Leisler, 1812) Temminck’s stint (Saheli teteeri)	Scolopacidae (Charadriiformes)	18	3	16	0	0	0	7
63	*Gallinago gallinago* (Linnaeus, 1758) Common Snipe (Drum Tel)	Scolopacidae (Charadriiformes)	10	3	3	0	1	0	8
64	*Tringa stagnatilis* (Bechstein, 1803) Marsh Sandpiper (Drum Tel)	Scolopacidae (Charadriiformes)	5	3	3	0	0	0	3

MD, medicinal; FD, food; SPS, superstitious; HN, hunting; EX, export; OR, ornamental

### Significant differences in the use of waterbird species

**Fig. 4:**
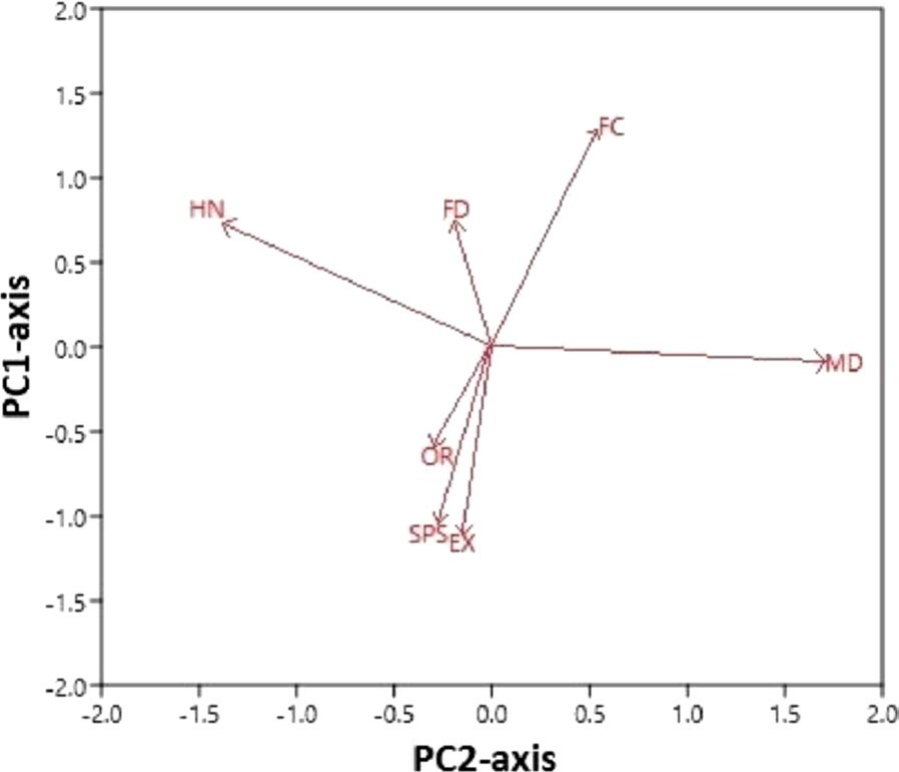
Principal component analysis of folklore data; codes are written in Table 1

**Fig. 5:**
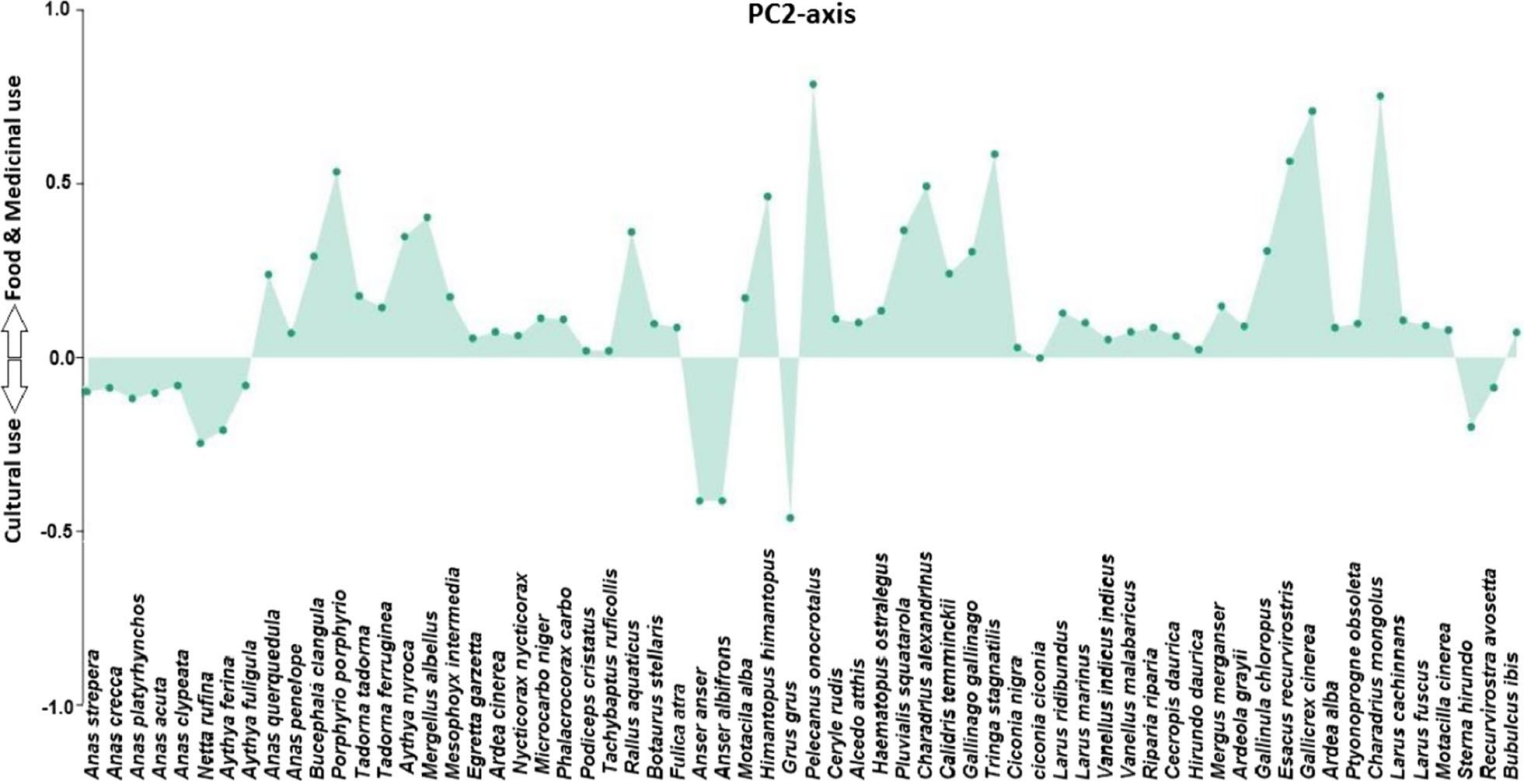
Loadings of variables in PC2-axis separating the cultural, food, and medicinal use of waterbird species

There were significant differences in the use of water bird species for cultural and medicinal purposes separated along the axis-2 (*p* < 0.05) as shown in Fig. 4. The significance of PCA scores was confirmed by one-way ANOVA, which calculated the analytic differences between cultural and medicinal use of waterbird species. PC1 and PC2 elucidated 86% of the variance in the PCA conducted with MD (medicinal), FD (food), SPS (superstitious), HN (hunting), EX (export), and OR (ornamental). Loadings of variables in PC2 showed that *Anas strepera, Anas crecca*, *Anas platyrhynchos, Anas acuta, Anas clypeata, Netta rufina, Aythya ferina, Aythya fuligula, Anser anser, Anser albifrons*, *Grus grus, Sterna hirundo, Recurvirostra avosetta,* and *Bubulcus ibis* were negatively correlated with cultural use value (Fig. 5).

### Quantitative assessment of medicinal waterbird species

#### Relative frequency of citation (RFC)

The highest value of “*relative frequency of citation*” is documented in mallard (*Anas platyrhynchos*) as 4.06, followed by Gadwall (*Anas strepera*) (3.87), Common Pochard (*Aythya ferina*) (3.82), and Great White Pelican (*Pelecanus onocrotalus*) (3.82) (Table 2).

**Table 2:** Ethnomedicinal uses of water bird species in the study area

Sr. No.	Scientific name	Local name	DIS	Code	BPU	MOA	Ailments	RFC	FAC	FL	RPL	ROP	Reported use	SI
1	*Anas strepera*	Khar Sari batha	WV	AGS	Meat	Oral	Paralysis, cold, male impotency	3.87	39	48.15	1.00	48.1		0
2	*Anas crecca*	Warri Chorak	WV	ACCT	Meat	Oral	Cough, cold, male impotency	3.77	42	53.16	1.00	53.2		0
3	*Anas platyrhynchos*	Sheen sar Batha	WV	APM	Meat	Oral	Cancer, cough, cold, male impotency, diabetes, BP piles, arthritis, body sickness during pregnancy, fever, heart problems, cut and wound, eye pain, TB	4.06	85	100	1.00	100	Fever, weakness, BP, cancer, weight loss, eye [12], paralysis, weakness [11, 64], erectile dysfunction [65], TB [66]	1
4	*Anas acuta*	Laki mar Batha	WV	ACNT	Meat	Oral	Male impotency, cough, cold,	3.72	39	50.00	1.00	50.0		0
5	*Anas clypeata*	Shabli	WV	ACS	Meat	Oral	Cough, cold, male impotency	3.34	36	51.43	1.00	51.4		0
6	*Netta rufina*	Shabar	WV	NRRP	Meat	Oral	Cough, cold, male impotency	3.53	14	18.92	1.00	18.9		0
7	*Aythya ferina*	Sor-sari Batha	WV	AFCP	Meat	Oral	Paralysis, male impotency	3.82	27	33.75	1.00	33.8		0
8	*Aythya fuligula*	Ziar Stergi Batha	WV	AFTD	Meat	Oral	Cough, cold, male impotency	2.00	9	21.43	0.99	21.2		0
9	*Anas querquedula*	Gergani	WV	AQG	Meat	Oral	Cough, cold, male impotency	3.20	45	67.16	1.00	67.2		0
10	*Anas penelope*	Seti mar Batha	WV	MPW	Meat	Oral	Cough, cold, male impotency	3.34	48	68.57	1.00	68.6		0
11	*Bucephala clangula*	Zangli Charga	WV	BC	Meat	Oral	Cough, cold, male impotency	2.63	17	30.91	1.00	30.9		0
12	*Porphyrio porphyrio*	Jamani Charga	R	PPH	Egg	Oral	Cough, asthma	0.72	5	33.33	0.35	11.8		0
13	*Tadorna tadorna*	Spena Batha	WV	TTS	Meat	Oral	Male impotency, cough, cold,	3.05	39	60.94	1.00	60.9		0
14	*Tadorna ferruginea*	Sorhab	WV	TTRS	Meat	Oral	Cough, cold, male impotency	3.20	36	53.73	1.00	53.7		0
15	*Aythya nyroca*	Seti mar wari Batha	WV	ANFD	Meat	Oral	Cough, cold, male impotency	1.81	32	84.21	0.89	75.3		0
16	*Mergellus albellus*	Spena Bata	WV	MASD	Meat	Oral	Cough, cold, male impotency	1.72	26	72.22	0.85	61.2		0
17	*Microcarbo niger*	Warri tori Heley	R	MNLC	Meat	Oral	Cough, body pain	2.63	36	65.45	1.00	65.5		0
18	*Phalacrocorax carbo*	Gati Tori Heley	R	BSEB	Meat	Oral	Cough, body pain	2.58	36	66.67	1.00	66.7		0
19	*Podiceps cristatus*	Ghat greb	WV	PCCG	Meat	Oral	Cold, cough, male impotency	2.72	21	36.84	1.00	36.8		0
20	*Tachybaptus ruficollis*	Warri Grab	R	TRLB	Meat	Oral	Cough, cold, male impotency	2.72	21	36.84	1.00	36.8		0
21	*Rallus aquaticus*	Khawar cherge	R	RAWR	Egg	Oral	Asthma	0.48	3	30.00	0.24	7.1		0
22	*Fulica atra*	Jal kokar	R	FAC	Meat	Oral	Male impotency, cold	3.63	54	71.05	1.00	71.1		0
23	*Anser anser*	Warri mergabi	WV	AAGG	Meat	Oral	Arthritis, body pain	3.48	9	12.00	1.00	12.3		0
24	*Anser albifrons*	Ghati mergabi	WV	AAG	Meat	Oral	Arthritis, body pain	3.48	9	12.00	1.00	12.3		0
25	*Himantopus himantopus*	Tor Tetare	R	HHBS	Meat	Oral	Kidney stone	0.38	3	37.50	0.19	7.1		0
26	*Pelecanus onocrotalus*	Kotanra	WV	POGP	Fat, skin	Oral	Arthritis, body Pain	3.82	79	98.75	1.00	98.8		0
27	*Pluvialis squatarola*	Kherari	WV	FSGP	Meat	Oral	Cold	0.67	5	35.71	0.33	11.8		0
28	*Charadrius alexandrinus*	Speni Kherari	WV	CASK	Meat	Oral	Cold	0.48	5	50.00	0.24	11.8		0
29	*Calidris temminckii*	Saheli Tetari	WV	CTTS	Bone	Oral	TB, kidney stone	0.86	3	16.67	0.42	7.1		0
30	*Gallinago gallinago*	Drum tel	WV	GGCS	Meat, bone	Oral	Piles	0.48	3	30.00	0.24	7.1		0
31	*Tringa stagnatilis*	Drum tel	WV	TSMS	Meat, bone	Oral	Piles	0.24	3	60.00	0.12	7.1		0
32	*Ciconia nigra*	Tor Zarhi	WV	CNBS	Meat, fat, skin	Oral	Male impotency, arthritis	1.10	9	39.13	0.54	21.2		0
33	*Ciconia ciconia*	Spen Zarhi	WV	CCWS	Meat, fat, skin	Oral	Male impotency, arthritis	1.34	7	25.00	0.66	16.5		0
34	*Mergus merganser*	Tor sar Bata	WV	MMCM	Meat	Oral	Respiratory disorder, body pain, male impotency	2.29	27	56.25	1.00	56.3		0
35	*Gallinula chloropus*	Obo Charga	R	GCCM	Egg	Oral	Asthma	0.38	2	25.00	0.19	4.7		0
36	*Esacus recurvirostris*	Ghatee kharare	R	ERGT	Meat	Oral	Body weakness	0.19	4	100	0.09	11.8		0
37	*Gallicrex cinerea*	Zanglai Charga	R	GCWC	Egg	Oral	Asthma	0.33	7	100	0.16	16.5		0
38	*Charadrius mongolus*	Warri Kharari	R	CMLP	Meat	Oral	Cold	0.24	5	100	0.12	11.8		0
39	*Sterna hirundo*	Kaaz/babozi	S	SHCT	Meat	Oral	Gastric ulcer, obesity	0.62	1	7.69	0.31	2.4		0
40	*Recurvirostra avosetta*	Loi mahoki tetara	S	RAPA	Meat	Oral	Male impotency, cold	0.95	2	10.00	0.47	4.7		0

DIS, distribution; WV, winter visited; R, resident; S, summer breeder; BPU, body parts used; MOA, mode of application; FC, frequency of citation; FAC, frequency of ailment citation; FC, fidelity level; RPL, relative popularity level; ROP, rank order priority; SI, similarity index

#### Relative popularity level (RPL)

**Fig. 6:**
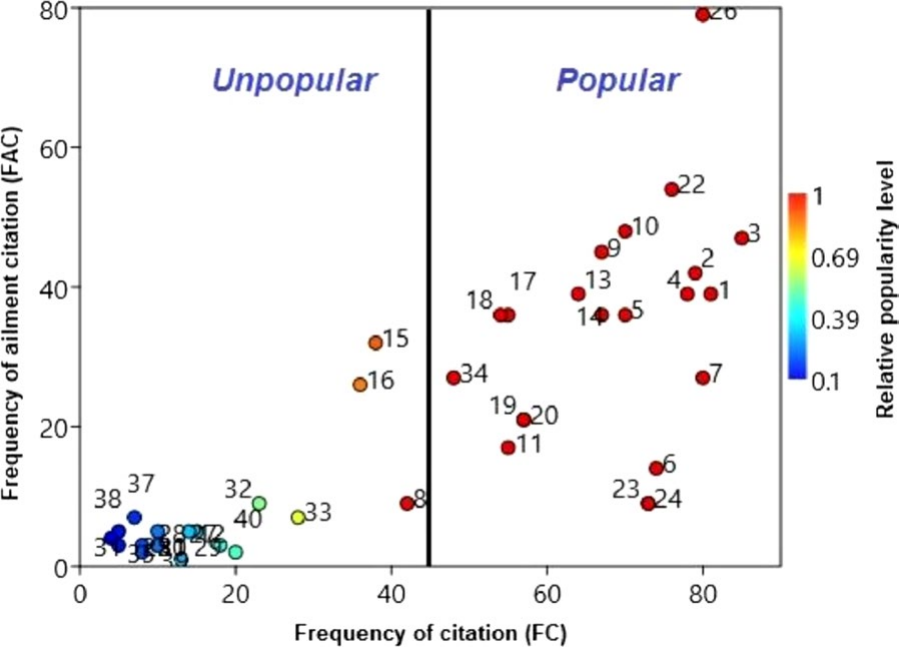
Relationship between numbers of informants (FC) claimed use of certain waterbird species for particular ailment (FAC). The species' relative popularity level (RPL) is determined and classified as popular or unpopular. Numbers represent the species names as they appear in Table 2

We documented 40 species that are used in ethno-pharmacological applications. Of total, 20 birds’ species, i.e., Gadwall, green-winged teal, mallard, northern pintail, northern shoveler, red-crested pochard, common pochard, garganey, Eurasian wigeon, common golden eye, common shelduck, ruddy shelduck, little cormorant, great cormorant, great crested grebe, little grebe, Eurasian coot, graylag goose, great white-fronted goose, great white pelican, and common merganser, were found more popular by respondents and have the highest “RPL” value (RPL = 1.00) (Fig. 6).

#### Fidelity level (FL%)

“*Fidelity level*” of waterbird species varied from 12 to 100%. A 100% “*fidelity level*” was calculated for only four waterbird species, i.e., mallard, great stone-curlew, watercock, and lesser sand plover. A total of 10 bird species showed an “*FL%*” value greater than 60%, i.e., common shelduck (60.94%), little cormorant (65.45%), great cormorant (66.67%), garganey (67.16%), Eurasian wigeon (68.57%), Eurasian coot (71.05%), smew (72.22%), ferruginous duck (84.21%), and great white pelican (98.75%) (Table 2)*.*

#### Rank order priority (ROP)

The “*rank order priority*” is utilized to determine the appropriate position of species of birds with different “*fidelity level*” values and the “*rank order priority*” (Table 2). In total, only 7 species attained a value of “*rank order priority*” *above 60*. These species are common shelduck (60.9), smew (61.2), little cormorant (65.5), great cormorant (66.7), garganey (67.2), Eurasian wigeon (68.6), Eurasian coot (71.1), ferruginous duck (75.3), and great white pelican (98.8) (Table 2).

## Discussion

### Socio-demographic data

Gathering socio-demographic data on participants (gender, age, educational level, occupation, and ethnicity) is particularly beneficial in ethnobiological research, as this element plays a significant role in analyzing and interpreting the responses received [67]. The older respondents, particularly those aged over 30 years, were highly populated in the study area (Fig. 2) and possessed significantly more traditional knowledge compared to younger participants. Community elders are often the holders of the most species information [68]. They are engaged in family responsibilities such as finance, health, and education and do not pass their knowledge to the next generation. As a result, knowledge of medicinal waterbird usage is diminishing. Similar research conducted in Pakistan and other countries showed that older respondents had significant traditional knowledge than younger participants [69–72].

Educated individuals in the study region were found to be less knowledgeable about the use of medicinal waterbirds than illiterate people, due to their higher exposure to modernization. Similar findings were reported in the research studies conducted in southern KPK [73] and central Punjab [11].

### Temporal shifts of folk knowledge and local nomenclature

According to traditional health practitioners (THPs), knowledge about the use of medicinal waterbird(s) was derived from either one or more of these sources: (i) medicinal knowledge regarding the use of waterbird(s) was passed from generation to generation within the family, (ii) folk knowledge was gained from teachers, religious scholars, and hakeems, (iii) knowledge was gained from reading published traditional folklore books, (iv) knowledge was obtained by experimentation with waterbird species, which was then applied on humans, (v) traditional knowledge was gained in aspirations, and (vi) a comparable assortment of medicinal waterbird(s) to treat any specific ailment of the human body parts. Transfer of cultural knowledge and traditional information from parents to children, preferably to sons, was found to be the most prevalent, as in other communities across the world [74–79]. Moreover, local taxonomy represents the vernacular names of species which give clues about social associations, myths, morphological differences, and ecology [80].

### Folklore and cultural applications of waterbird species

**Fig. 7:**
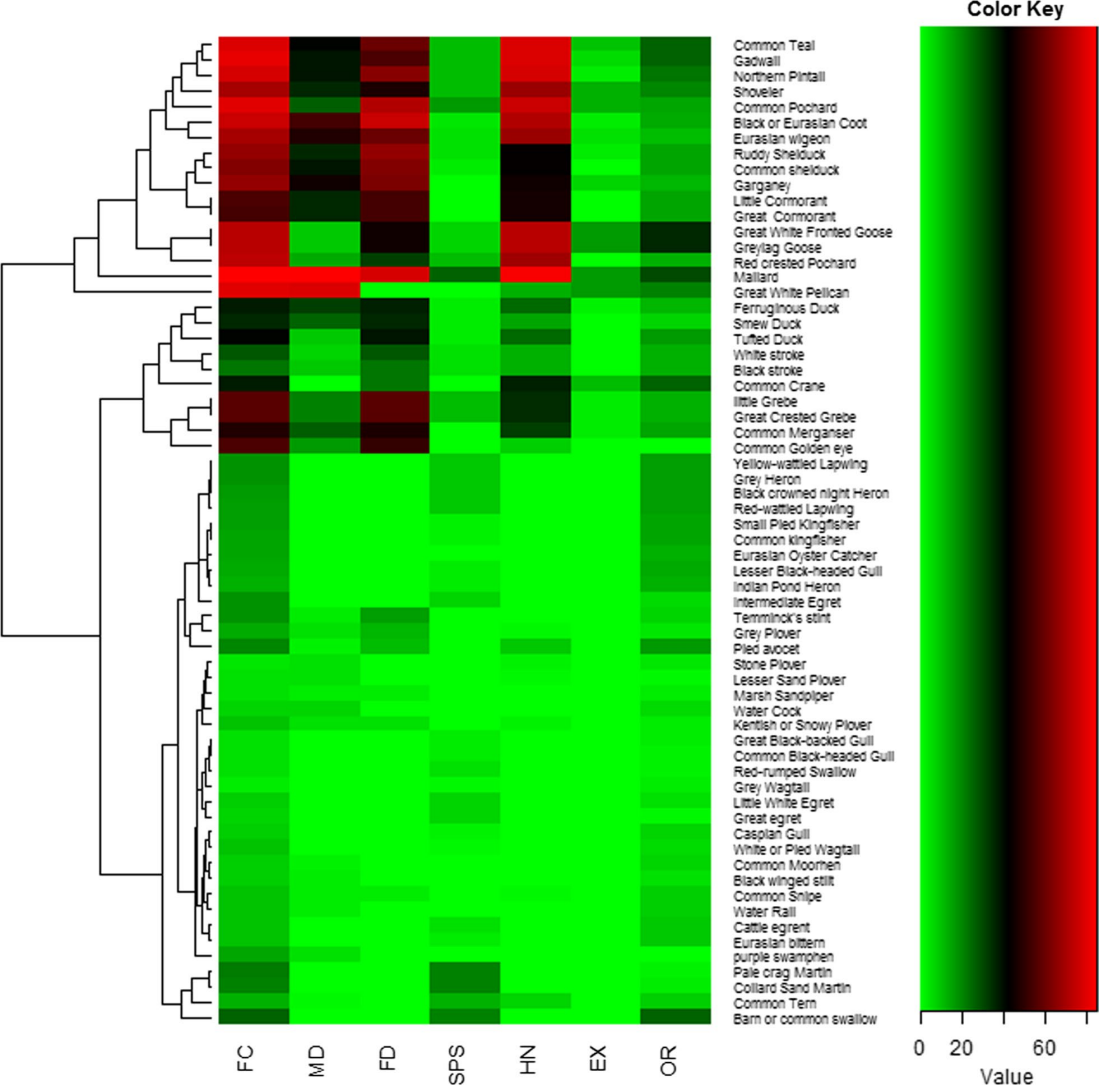
Heatmap of waterbird species usage by informants for MD (medicinal), FD (food), SPS (superstitious), HN (hunting), EX (export), and OR (ornamental) purposes in Eastern KPK, Pakistan. Green and red colors indicate increased and decreased values of informants, respectively

Some waterbird species are more used as food, medicine, and hunting, e.g., mallard, common teal, gadwall, northern pintail, shoveler, common pochard, Eurasian coot, Eurasian wigeon, garganey, great white-fronted goose, graylag goose, little cormorant, great cormorant, and red-crested pochard (Fig. 7). In total, 40 waterbird species were utilized as foodstuffs in the study area (Table 1). A total of 18 species are exported from the study area, while the feathers of 62 waterbird species are utilized in decoration (Table 1 and Fig. 7). Waterbird species are also utilized as food, according to other ornithologists [2, 3, 11].

Forty-four species of birds are connected with superstitious beliefs, such as people of the local area believing that ducks (i.e., gadwall, common teal, mallard, northern pintail, northern shoveler, red-crested pochard, common pochard, tufted duck, garganey, European wigeon, common shelduck, ruddy shelduck, ferruginous duck, smew, great crested grebe, Eurasian coot, and little grebe), kingfishers (i.e., pied kingfisher and common kingfisher), and gooses (i.e., graylag goose and great white-fronted goose) are a sign of prosperity. The following are superstitions about egrets (i.e., intermediate egret, little white egret, cattle egret, and great egret) and terns (i.e., black-bellied tern and common tern): If someone harms egrets, it will be bad luck (i.e., gray heron, Indian pond heron, and black-crowned night heron). It is documented that herons are a sign of bad luck if they are present at home. Superstition about storks (black stork and white stork) is that when storks lay down their heads and necks back over their bodies at this time, it means a storm will come. Gulls are also superstitious in the study area, as if three gulls (i.e., common black-headed gull, Caspian gull, lesser black-headed gull, and great black-backed gull) are flying directly over a person; it is a sign of the death of this person. Likewise, it is noted that if red-rumped swallows and martens (i.e., sand martin and pale crag martin) are settled in any house, it is a sign of poverty. Similarly, lapwings (yellow-wattled lapwing and red-wattled lapwing) have superstitions that if these birds cry at your house, it is a sign of a visitor (Table 1). These findings were also documented by other ornithologists [5, 11, 81].

### Ethnomedicinal uses of waterbird species

**Fig. 8:**
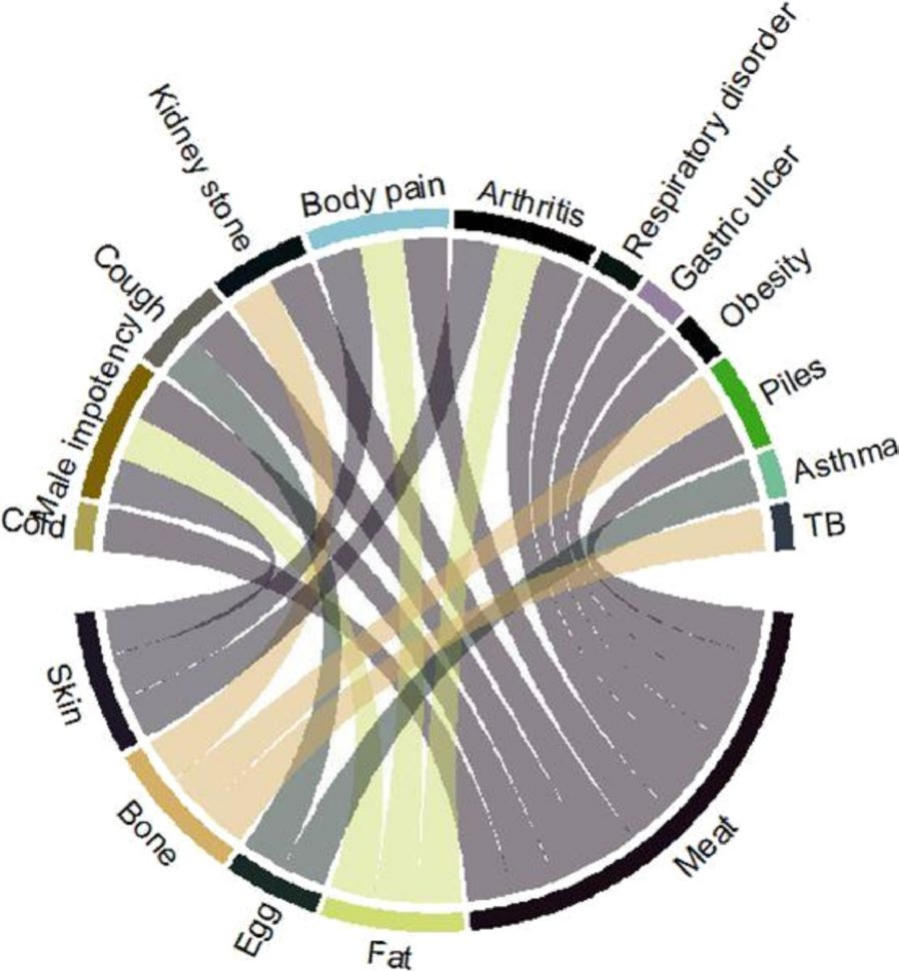
Relationship between body parts and diseases used in the study area

**Fig. 9:**
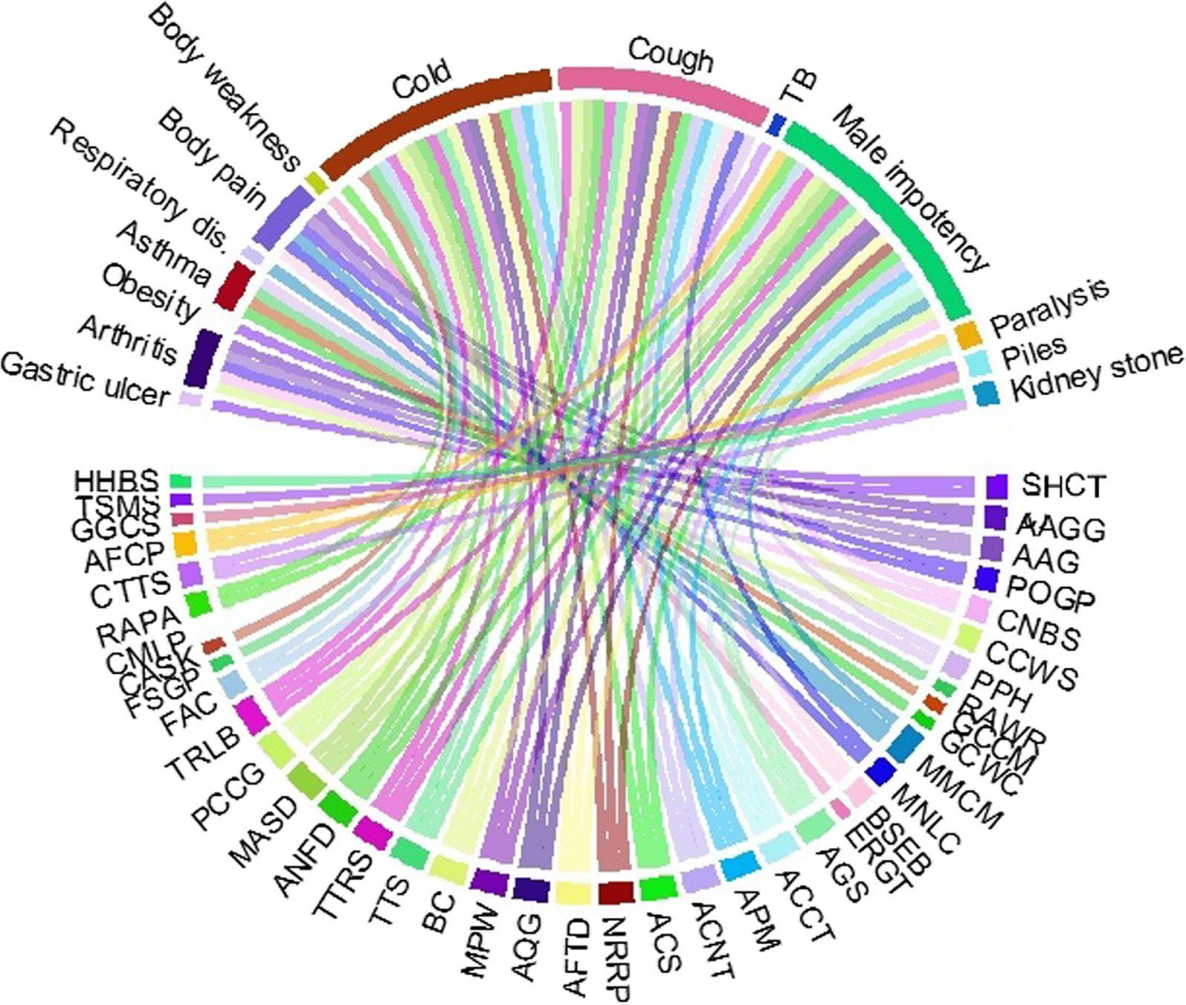
Waterbird species distribution according to the treatment of various ailments in Eastern KPK, Pakistan. Codes represent the species names as they appear in Table 2

The meat of waterbird species was the most utilized body part in the study area (Fig. 8). Meat of waterbird species was commonly used to treat various human ailments such as respiratory disorders, gastric ulcers, arthritis, obesity, body pain, and piles (Fig. 9). People specifically hunted waterbirds for meat. Cold, cough, fever, flu, bronchitis, breathing problems, infertility, asthma, abscess, anemia, body weakness, body strength, enhanced memory, immune enhancer, epilepsy, menorrhagia, paralysis, puberty in young girls, skin diseases, sexual power, and wound healing are all treated with meat from various waterbird species [1, 3, 7, 17, 28, 82–86]. The inhabitants of the study area also use fat to treat arthritis, body pain, and male impotency (Table 2 and Fig. 8). In fact, the presence of “omega-3 fatty acid” in fat cures inflammation [87]. Moreover, “omega-3 fatty acid” is also useful in atherosclerosis, thrombotic, neurological disorders, and aging effects [10, 88–90].

It was found that local inhabitants of the study area used various waterbird species to treat different infectious and chronic diseases like cold, cough, flu, fever, respiratory disorders, asthma, TB, gastric ulcers, kidney stones, male impotency, obesity, paralysis, piles, cancer, arthritis, body pain, and weakness (Fig. 9). Other studies also reported that waterbird species were used to treat respiratory disorders (asthma, pneumonia, and cough), cardiovascular disorders, and skin infections [11, 91]. Moreover, waterfowl are a major part of the diet of indigenous people at high latitudes in North America [92]. The main reasons for the higher number of diseases in this remote area might be a lack of exercise, nutritional deficiency, and a polluted environment. However, THPs are more familiar with the use of parts and products of waterbird species for the treatment of various human ailments. Some of the local inhabitants hunted bird species and sold them in local markets or to hakeems, normally at low prices. THPs use the products or parts of waterbird species in suitable seasons or at specific times. Many THPs kept written notes for medicinal preparations but usually did not share such information publicly, so as not to increase the number of practitioners.

The separating line between the popular and unpopular groups falls at the point where the average number of uses per species ceases to increase with a further increase in the number of informants (Fig. 8). Based on the RPL index analysis, we found certain popular species that are utilized to cure a greater number of diseases in the study region, i.e., mallard, gadwall, green-winged teal, garganey, Eurasian wigeon, and Eurasian coot. The high popularity of these plant species might be attributed to their high efficacy which specifies their use as therapeutic medicine. Moreover, 100% FL was noted for four waterbird species, i.e., *Charadrius mongolus* (cold), *Gallicrex cinerea* (asthma), *Anas platyrhyn*chos (cancer), and *Esacus recurvirostris* (body weakness). Mainly, waterbird species with 100% FL are utilized more in the traditional healthcare system of the study area [93, 94]. The high familiarity of waterbird species might be recognized by their wider distribution, diversity, and familiarity with the people of the study area, which specifies their use in ethno-pharmacological applications. These findings are supported by other ethnobiologists [56, 61]. Waterbird species with high RPL and FL values showed the importance of these species and are proposed for further pharmacological evaluation to analyze their therapeutic potential and for screening of unknown bioactive chemicals.

#### Critical analysis of medicinal waterbird species

The ethno-pharmacological data were calculated using PCA, which assigned the six variables for the ordination of designs in terms of MD, FD, SPS, HN, EX, and OR. It is clear from our results that local residents used the waterbird species more for medicinal and food purposes (Fig. 5). Previous results showed that wild birds are used as a source of food in many areas of the world, i.e., India [95, 96], Pakistan [11, 28], Philippines [97], and Brazil [91, 98]. However, statistical analysis is highly valuable in ethnobiological studies because it provides important information for pharmacological and clinical studies.

Waterbird species are used to treat different human ailments, which reflects that the people of Eastern KPK have more information to control the healthcare system and that traditional pharmacological applications have not been eliminated from the culture. The high usage of waterbird species may be due to the abundance and widespread dispersion of these species in the study area. Furthermore, traditional medicine for curing various ailments may also result in high RFC, RPL, and FL [99–101]. In this study, mallard was the most popular species in Eastern KPK with high FL (100%), which show the abundance and wider use of this species' by-products for cancer treatment (Table 2). In their study, Altaf, Umair [12] reported that mallard was used to treat cancer by the local communities of Punjab, Pakistan.

**Fig. 10:**
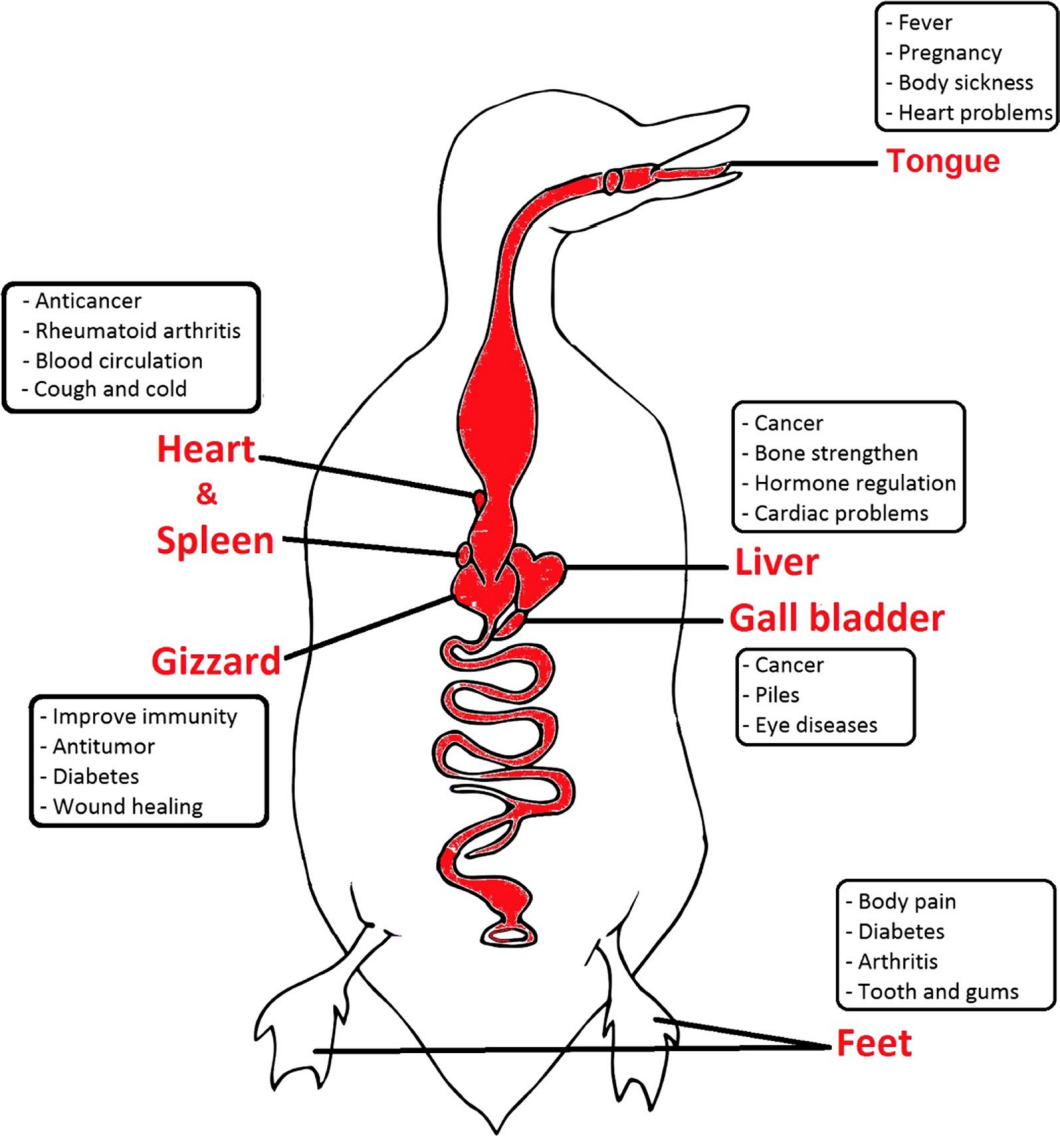
Graphical representation of the medicinal uses of mallard (*Anas platyrhynchos)* in Eastern KPK, Pakistan

Most wild duck by-products, such as liver, gizzard, heart, and spleen (Fig. 10), are rich sources of essential nutrients and polyunsaturated fatty acids [102]. In comparison with other tissues, El-Sayed, Farag [103] found that the liver and gizzard are the best sources of high-quality protein. A high-protein diet has been demonstrated to boost metabolism, control appetite, and enhance muscle growth and preservation during weight reduction [104, 105]. Despite this, it is also high in minerals and vitamins, including copper, vitamin A, and several essential amino acids [106]. Trace elements are also known as microelements and are essential for bone formation, hormone production, and heart rate regulation [107, 108]. Furthermore, all of the by-products, particularly the liver, had larger quantities of microelements (e.g., Cu, Fe, Mn, and Zn) than muscle tissues [109]. Copper (Cu) is an essential microelement, and the human body requires only a minimal amount [108]. According to Garber [110], copper has higher antioxidant properties and can help to fight cancer.

Liver and fat are used to treat swelling wounds and pneumonia [66], influenza, bronchitis, asthma [111], blisters, and skin problems [112, 113]. Duck tongue meat is said to be especially beneficial to people recovering from illness and to alleviate body sickness during pregnancy. In another study, duck bile is used to treat cancer, traumatic hemorrhage, and dyspepsia [114, 115]. Likewise, duck gizzard peptides can provide a plentiful source of natural antioxidants for applications in the food industry [116]. The gizzard is a low-fat, high-protein organ that has great natural levels of iron and zinc [103]. These nutrients support a healthy immune system, promote wound healing, and aid in cell division. The dark-colored large duck hearts are very low in calories, and in terms of their nutritional value, they are as good as the hearts of other animals [117]. Both the heart and spleen are rich in protein and saturated fatty acids, which are helpful in improving blood circulation and curing cancer, cough, cold, and rheumatoid arthritis [118]. Duck feet are a natural source of glucosamine, chondroitin, and collagen [119], which provide joint health by producing joint fluid, reducing the risk of brittle bones, improving mobility, and helping maintain healthy teeth and gums.

### Bio-conservation or sustainable use of the reported species

For the design and integration of biodiversity conservation plans, understanding the knowledge of human–animal interaction and the use of natural resources is critical [120]. However, the documentation of indigenous knowledge on animal-based medicines is very helpful in the formulations of strategies for sustainable management and conservation of bio-resources [121]. Ethno-ornithological studies, in addition to integrating biological factors and giving traditional knowledge on medicinal values of species in any region, also cover social, economic, traditional, and cultural values of animal species in human societies and thus make a significant contribution in animal conservation [26].

Use of waterbird species in traditional therapies and for cultural purpose by humans is not the only threat to bird diversity in any region. Factors also include changes in climate and various types of interactions in an ecosystem, i.e., food chain and food webs also contribute significantly to threatening waterbird population and diversity [26, 34]. Given the great need to find solutions to deal with the current crisis of biodiversity loss [122], more specifically that of bird species, it is obligatory to adopt strategies that address the problem in all its complexity. And for this, ethnozoology presents itself as an interdisciplinary tool, approaching the issue in an additional comprehensive method [123].

### Novelty of the study

The current study is a collective effort that includes both documenting and cross-cultural comparisons of the reported species in order to better understand the different waterbirds usage traditions. We found a high degree of overlap in the use of specific waterbirds among ethnic groups. Because of their food value, certain species were found to be more prevalent in all cultures. Moreover, the collected data are unique because these waterbird species have no previous records. We found that all waterbird species have a 0.00 “similarity index.” Only 1 species (i.e., mallard) has a 1.00 similarity index and has been reported for ethnomedicine applications previously. In the current study, this species was used to treat cancer, cough, cold, male impotency, diabetes, BP piles, arthritis, body sickness during pregnancy, fever, heart problems, cut, wounds, eye pain, and TB, while in reported use, this species was used to cure fever, weakness, colds, BP, cancer, weight loss, eye pain [12], paralysis, weakness [64], erectile dysfunction [65], and TB [66].

## Conclusion

To treat human ailments, the local inhabitants of Eastern KPK used 40 species of waterbirds. The present collected data showed that a lot of medicinal waterbird species are used by confined societies. The native people still rely on traditional medicine in Eastern KPK instead of the presence of other healthcare departments; thus, medicinal waterbird species have significant value in treating a variety of human ailments. Compiled data showed that high RFC, FL, RPL, and ROP values showed that popular waterbird species are the most preferred for specific ailments. These results could be helpful for the sustainable use of waterbird species in the traditional healthcare system. However, the main threats to the diversity of waterbirds in the area are hunting, trading, and cultural use.

## Data Availability

All the data are presented in tables and figures in the article or as supplementary material, and further inquiries can be directed to the corresponding authors.
